# Weight Is a Predictor of Delayed Operation Time in Primary Isolated Anterior Cruciate Ligament Reconstruction

**DOI:** 10.3390/biomedicines11082137

**Published:** 2023-07-29

**Authors:** Sungtae Lim, Sung-Sahn Lee, Juyong Oh, Dae-Hee Lee

**Affiliations:** 1 Department of Orthopaedic Surgery, Samsung Medical Center, Sungkyunkwan University School of Medicine, Seoul 06351, Republic of Korea; lst0807@gmail.com (S.L.); dhwndyd007@naver.com (J.O.); 2 Department of Orthopaedic Surgery, Ilsan Paik Hospital, Inje University School of Medicine, Goyangsi 10380, Republic of Korea; sungsahnlee@gmail.com

**Keywords:** anterior cruciate ligament, reconstruction, operation time, weight, body mass index, complications

## Abstract

Background: Few studies have evaluated the impact of obesity on operation time in patients with ACL reconstruction. The purpose of this study was to understand the effect of obesity on operation time in patients with arthroscopic anterior cruciate ligament (ACL) reconstruction. Methods: A total of 103 patients were included. The mean pure operation time was 45.9 ± 13.4 min. Considering that 15 min incremental increases in operation time are an independent risk factor for complications, all patients were classified into two groups according to operation time: more or less than 61 min. Demographic data were compared between both groups. Pure operation time was defined as operative time without suture time (pure operation time = suture start time − operation start time). Correlation analysis between demographic data and pure operation time was performed, and multiple linear regression analysis was used to identify the predictors of pure operation time. Results: The pure operation time ≥61 min group (*n* = 34) had a 14.7 kg higher weight and 4.5 kg/m^2^ higher body mass index (BMI) than those with pure operation time < 61 min (*n* = 69). Weight (r = 0.635, *p* < 0.001) and BMI (r = 0.584, *p* < 0.001) were positively correlated with operation time. Multiple linear regression analysis showed that weight (β = 0.635, *p* < 0.001) was the only predictor of operation time. A weight of 74.25 kg was a cut-off value for a pure operation time of >61 min. Conclusions: The weight and BMI of the group with pure operation time of ≥61 min were 14.7 kg and 4.5 kg/m^2^ higher, respectively. The weight of patients with ACL tears was a factor affecting delay in the operation time. Patients weighing over 74.25 kg were more likely to delay ACL reconstruction.

## 1. Introduction

The knee joint is a hinge joint, which comprises two articulating bones: the tibia and femur. The static stability of the knee joint depends on the ligaments. Among the ligaments of the knee joint, the anterior cruciate ligament (ACL) is not only important for preventing anterior translation of the tibia over the femur, but it is also crucial for knee joint rotatory stability [1,2]. ACL is primarily involved in knee joint pivoting, jumping or running, and it is the most common ruptured ligament in the knee joint during sports activity. Therefore, ACL reconstruction is one of the most common orthopedic arthroscopic procedures performed worldwide [3]. According to United States national data, there were approximately 125,000 ACL reconstructions performed in 2006 [4]. A more recent study demonstrated that the incidence of ACL injury is approximately 29–78 per 100,000 person years [5]. The incidence of ACL tears has been increasing yearly, and the rate of ACL reconstruction is projected to continue to rise annually [6].

Given these trends, it is important to identify modifiable risk factors for complications or readmission following ACL reconstruction because these unexpected situations could increase the burden not only on the patients and surgeons but also on health repayment systems. It is widely known that short-term complications increase as the operation time increases in various orthopedic procedures. Surgical time has previously been reported to be an identified risk factor for postoperative complications, including extended length of hospital stay and hospital readmission rated after total knee arthroplasty, total hip arthroplasty or shoulder joint arthroscopic surgeries [7,8,9]. It is also well known that the more the short-term complications occur after ACL reconstruction, the longer the operation time; therefore, reducing the ACL reconstruction operation time is a very important orthopedic concern [10]. Previous studies suggested that operative length greater than 1 h could increase the risk of surgical site infections or thromboembolism after ACL reconstruction [11,12].

Obesity is a serious public health problem, which is increasing in prevalence in adults and children, with a disproportionate number of obese patients presenting for elective orthopedic surgery. It is generally accepted that obese patients are more likely to have worse postoperative outcomes. One reason for this unsatisfactory result after surgery is the increasing operation time due to obesity [9,13,14].

The correlation between prolonged operation time and obesity is well documented in the joint arthroplasty literature, with obese patients showing increased operation times and readmission rates within 30 days of total joint arthroplasty [15,16,17,18]. Nevertheless, relatively few studies have evaluated the impact of obesity on operation time in patients with ACL reconstruction.

Therefore, the purpose of this study was to understand the effect of obesity on operation time in patients with arthroscopic ACL reconstruction. It was hypothesized that obesity would lead to longer operation times in arthroscopic ACL reconstruction.

## 2. Materials and Methods

### 2.1. Study Design and Patients

This study is a retrospective study of a prospectively corrected ACL dataset in our institution. The study included 309 patients (311 knees) who underwent ACL reconstruction between April 2015 and June 2022 at our institution. The inclusion criteria consisted of patients who underwent pure ACL reconstruction with allograft tendon using the anteromedial portal technique. The exclusion criteria included patients who underwent a meniscal procedure (162 knees), received combined posterior cruciate ligament reconstruction (11 knees), revision of ACL reconstruction (4 knees), or received autografts (29 knees). All surgeries were performed at a single institution. The study design was approved by our institutional review board, and informed consent was obtained from each patient (SMC2022-11-081).

**Figure 1:**
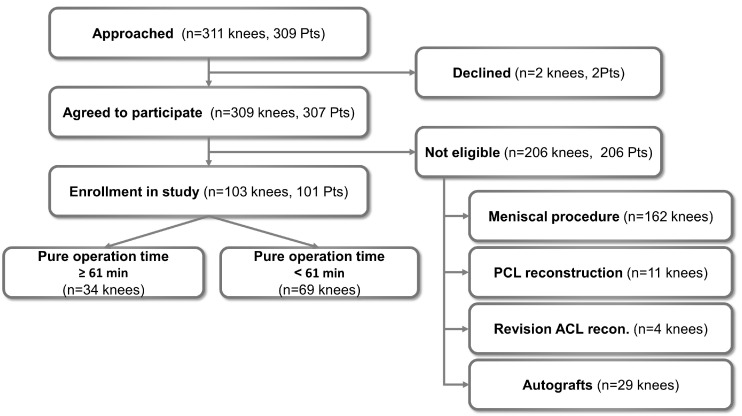
Flow chart describing the patient enrollment process in the study. Abbreviations: Pts, patients; recon., reconstruction.

It is common knowledge that 15 min incremental increases in operation time are an independent risk factor for complications. Moreover, an operation time longer than 1 h could increase the risk of surgical site infections after ACL reconstruction, as described previously [10,11]. Therefore, we classified all patients into two groups according to operation time of more or less than 61 min. The duration of 61 min is 15 min plus the mean of pure operation time (except suture time) of the present study data. Of the 309 patients (311 knees) initially approached for the study, 307 (309 knees) agreed to participate. After assessment for study eligibility, 101 patients (103 knees) were included in the final analysis. A total of 34 and 69 knees had pure operation times of ≥61 min and <61 min, respectively (Figure 1).

### 2.2. Surgical Technique

All surgeries were performed by a single senior surgeon, one of the authors (D.H.L) at a single institution, and the same surgical technique was applied for every patient. ACL reconstruction using the anteromedial portal technique requires two additional portals: the high and low anteromedial portals. The high anteromedial portal was placed at the mid-patellar level, and the low anteromedial portal was created along the medial border of the patellar tendon, entering just above the anterior horn of the medial meniscus.

After diagnosing an ACL injury, a femoral tunnel was created. The transanteromedial (AM) portal method was used to create the femoral tunnel at the anatomical position. The femoral footprint was marked using a radiofrequency device, while the target point was viewed through the high anteromedial portal. A Bullseye femoral guide (Linvatec, Largo, FL, USA) was used to penetrate the low anteromedial portal; a guide pin was inserted through the guide, and its tip was inserted into the previously marked footprint center. If the femoral footprint was not well visualized, as in chronic ACL tears, the guide pin was located below the lateral intercondylar ridge and slightly posterior to the lateral bifurcate ridge to mimic the anteromedial bundle. The femoral tunnel was created by reaming along the inserted guide pin with the knee at more than 120° of flexion. The tibial tunnel was subsequently drilled using a graft-sized reamer. The hamstring tendon graft was fixed with an extracortical EndoButton (Smith & Nephew Endoscopy, Andover, MA, USA) on the femoral side. Tibial fixation was performed using biodegradable interference screws.

Crutch-assisted walking was initiated 1 day after surgery. Full-weight-bearing walking was permitted at 6 weeks. Range of motion (ROM) exercises were started from 0° to 90° 2 days after surgery, and full flexion was achieved by 6 weeks. Closed kinetic chain exercises were started 2 weeks postoperatively. Sports activity, including pivoting, jumping or side-stepping, was allowed 9 months postoperatively.

### 2.3. Evaluation of Pure Operation Time

**Figure 2:**
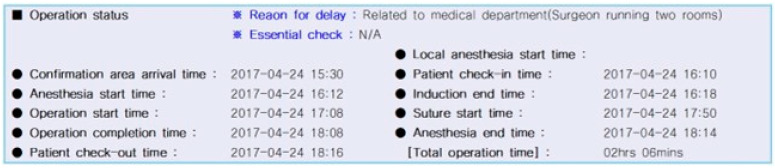
Timetable of operation record.

We evaluated the operation time using the operation timetable of the operation records (Figure 2). The operation timetable has many categories, including the total operation start time, suture start time and operation completion time. Patient check-in time was defined as the time the patient entered the operating room. The anesthesia start time was defined as the time at which the anesthesiologist started induction. The induction end time was defined as the time at which the anesthesiologist ended induction. The operation start time was defined as the time at which the surgeon started the first incision. The suture start time was when all procedures were completed, and suturing was started. The operation completion time was defined as the time at which all sutures were finished. The anesthesia end time was defined as the time when the anesthesiologist woke up the patient after anesthesia was completed. Patient check-out time was defined as the time when the patient was out of the operating room. Total operation time was defined as the duration between patients’ check-in and check-out times.

All ACL reconstructions in this study were performed by only one expert surgeon, except for suturing. Generally, sutures were performed by fellow doctors. To achieve a more accurate operation time by excluding bias due to suture time, we calculated pure operation time by excluding suture time (pure operation time = suture start time − operation start time). This is the pure operation time during which the surgeon wholly performed the operation, which included creating portals, femoral footprint marking, creating a femoral tunnel, creating a tibial tunnel, graft passage and graft fixation at the femoral and tibial sides. The patient distribution and evaluation of demographic data categorized by operation time are summarized in Table 1.

### 2.4. Statistical Analysis

A power analysis was performed to determine the sample size adequate for detecting a significant weight difference between groups with a pure operation time ≥ 61 min and <61 min. In a pilot study of 15 cases, 11 were classified as having a pure operation time ≥ 61 min and 4 as having a pure operation time < 61 min, with a 12 kg weight difference between the two groups. To detect a 12 kg difference between the two groups with a power calculation of 0.8, the required sample size was determined to be 25 for each group. The current study involved 34 and 69 cases of groups with operation times ≥ 61 min and <61 min, respectively. Overall, the power of the present study was 0.836 for detecting a 12 kg difference between the two groups.

The demographic data and operation time measurements were compared between groups with pure operation time (except suture time) ≥ 61 min and <61 min using Student’s t-test. The correlation coefficients between the operation time and demographic data were analyzed using Pearson’s correlation analysis. Multiple linear regression analysis was used to identify the independent variables, which affected operation time. Statistical analysis was performed using IBM SPSS Statistics version 27 (IBM Corporation, Chicago, IL, USA). All data are presented as mean and standard deviation. A *p*-value less than 0.05 was considered statistically significant.

To evaluate the cut-off value of weight for ≥61 min pure operation time, receiver operating characteristic (ROC) analysis (Medcalc version 19.0.7, MedCalc Software Ltd., Ostend, Belgium) was used. The ROC curve is a plot of sensitivity versus specificity of a test. The Youden index was used to assess the cut-off value of weight for a pure operation time of ≥61 min. This is one of the main statistics of the ROC curve, which defines the maximum potential effectiveness of a test: the test’s differentiating ability when equal weights are assigned to sensitivity and specificity [19].

## 3. Results

The patient distribution and evaluation of demographic data were categorized into two groups (pure operation time ≥ 61 min versus <61 min) and are summarized in Table 1.

Across all patients, the average age at surgery was 31.9 ± 12.6 years (range, 14–60 years); the mean weight was 72.4 ± 12.7 kg (range, 45.1–104.3 kg), and the mean body mass index (BMI) was 24.9 ± 4.1 kg/m^2^ (range, 17.1–36.0 kg/m^2^). The mean operation time was 62.5 ± 10.6 min, and the mean pure operation time (except suture time) was 45.9 ± 13.4 min. The pure operation time ≥ 61 min group (*n* = 34) had a 14.7 kg higher weight and 4.5 kg/m^2^ higher BMI than the pure operation time < 61 min group (*n* = 69, Table 1). Among demographic parameters, including age, height, weight and BMI, weight (r = 0.635, *p* < 0.001) and BMI (r = 0.584, *p* < 0.001) were positively correlated with the pure operation time (Table 2), although there was no correlation between demographic parameters and operation time, including suture time. However, multiple linear regression analysis showed that weight (β = 0.635, *p* < 0.001) was the only predictor of operation time (except suture time), and BMI was not a significant factor affecting operation time (β = 1.195, *p* = 0.16, Table 3).

**Figure 3:**
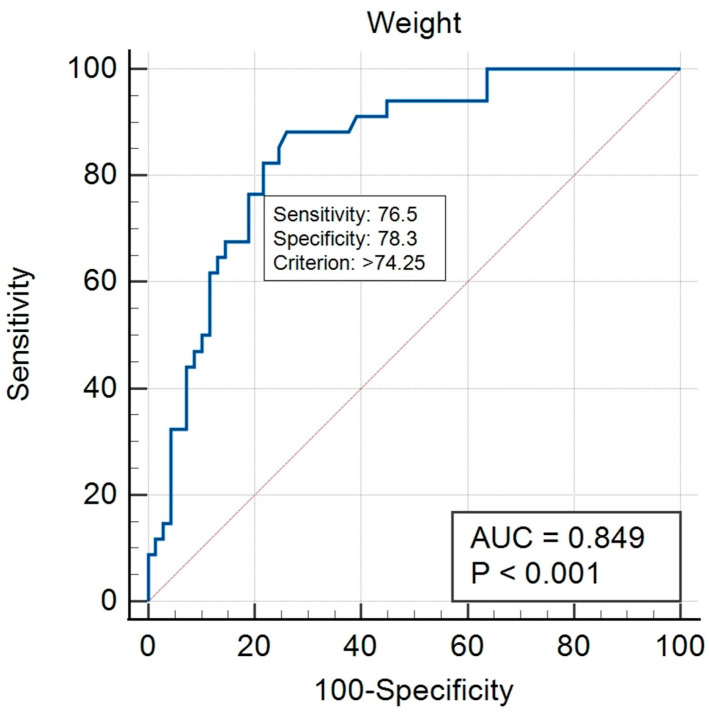
Receiver operating characteristic curves illustrating the weight cut-off values for predicting more than 61 min pure operation time (except suture time).

ROC analysis was performed to determine the threshold values for weight in terms of predicting a pure operation time ≥ 61 min. The area under the curve (AUC) for the weight of the pure operation time ≥ 61 min group was 0.849 (95% confidence interval [CI], 0.765–0.912). A weight of 74.25 kg was found to be the cut-off value for the operation time ≥ 61 min group, with a sensitivity of 76.5% and a specificity of 78.3% (Figure 3).

## 4. Discussion

The most important finding of the present study was that increased weight of patients with ACL tear predicted a more prolonged pure operation time in primary ACL reconstruction using the anteromedial drilling technique.

It is well established that obesity has a significant impact on orthopedic operation time [20,21,22]. Traven et al. conducted a retrospective analysis of 917 patients with ACL reconstruction registered in the National Surgical Quality Improvement Program (NSQIP). They found that BMI significantly predicted operation time; each additional BMI point led to an increase in operation time of 0.967 min or 58.0 s [23]. However, the patients included in that study were all adolescents, ranging from 14 to 17 years old, and 401 (43.7%) patients required additional procedures, such as meniscectomy or meniscus repair, at the time of ACL reconstruction. In addition, the NSQIP does not differentiate between primary and revision ACL reconstructions when registering data. Revision ACL reconstruction is a technically demanding procedure, which is inherently associated with prolonged operation time [24,25]. Therefore, the result of their study did not represent the ordinary case of isolated primary ACL reconstruction. Ballal et al. performed a single-center study, which compared the operation time after primary ACL reconstruction in a normal BMI group (*n* = 49, BMI < 25) versus a high BMI group (*n* = 43, BMI ≥ 25) [26]. The result of that study showed that the high BMI group had an 8 min longer operation time than the normal BMI group, although this was not statistically significant [27]. However, the cases in that study were also not isolated ACL tears, with 67% of the included patients (62/92) having associated injuries, such as in the meniscus or cartilage. Therefore, that study also has limitations in terms of assessing the relationship between obesity and operation time in isolated primary ACL reconstruction. Rather, the present study has advantages over the two aforementioned studies. All patients in our study had pure isolated primary ACL tears because we excluded patients with concomitant meniscus or cartilage injury and revisional ACL reconstruction. In addition, all surgeries for the included patients were performed by a single expert surgeon at a single hospital. Taken together, the results of our study could far more accurately reflect the operation time of pure isolated ACL reconstruction without any associated injury, such as meniscus or cartilage tear. In addition, our study reduced the variation bias due to the lack of generalization of the ACL reconstruction technique by different surgeons in different hospitals.

It is generally accepted that knee arthroscopy is far more technically challenging in overweight patients [28,29]. The difficulty in establishing arthroscopic portals in patients with obesity is a well-known concern for arthroscopic knee surgeons. In patients with obesity, it is difficult to palpate the bony landmarks required for anatomic positioning of the arthroscopic portals because of the large amount of superficial adipose tissue. These thick subcutaneous adipose tissues can narrow the operative and visual fields. Furthermore, our study used the anteromedial portal femoral drilling technique, which usually requires deep knee flexion greater than 120° during femoral tunnel preparation [30,31]. The anteromedial portal femoral drilling technique itself could cause arthroscopic apparatus crowding in the operation field and block the arthroscopic view [32,33,34]. This condition could be much worse in patients with obesity than in normal-weight patients. This situation could explain, to some extent, the reason why a longer operation time was required in patients with obesity than in normal-weight patients when performing ACL reconstruction in our study.

Surgical time is a correlated factor in postoperative complications, including extended length of hospital stay and hospital readmission rated after total knee arthroplasty, total hip arthroplasty or shoulder joint arthroscopic surgeries [7,8,9]. Belmont et al. had demonstrated that patients who performed total knee arthroplasty with operating times over 135 min had a significantly higher rate of complications, including pulmonary embolism, urinary tract infection, postoperative sepsis and deep venous thrombosis [8]. Bohl et al. had reported that incremental 15 min increases in operative time during lower extremity total joint arthroplasty induced 9% and 13% increases with respect to surgical site infections and wound dehiscence, respectively [13]. Boddapati et al. had investigated the effect of shoulder arthroscopy time on postoperative complications, readmissions and overnight hospital stays by analyzing 33,095 cases [9]. They concluded that increased shoulder arthroscopy procedure time is correlated with adverse short-term outcomes, especially superficial surgical site infection and overnight hospital stay. These finding could also be applied to ACL reconstruction. McAllister et al. had demonstrated that concurrent meniscal repair induced increasing infection rates following ACL reconstruction [35]. Westermann et al. also reported that postoperative infections were more prevalent in patients with concurrent collateral ligament surgery or those undergoing tibial osteotomy [11]. Moreover, previous studies suggested that operative length greater than 1 h could increase the risk of surgical site infections or thromboembolism after ACL reconstruction [11,12].

It is also well known that the more the short-term complications occur after ACL reconstruction, the longer the operation time; therefore, reducing the ACL reconstruction operation time is a very important orthopedic concern [10].

Our study suggested that the possibility of 15 min prolongation of the average operation time can be predicted preoperatively based on the weight of patients. We considered the 15 min excess from the average operation time as an important point because a previous study reported that there was a higher complication rate in ACL reconstruction, including deep vein thromboembolism or infection, in cases where the operation time was greater than 15 min over the average operation time [10]. The analysis of the ROC curve in this study found that a weight greater than 74.25 kg is the cut-off point value, which has the possibility of surpassing operation time by more than 15 min from the average operation time. This result indicates that when performing isolated ACL reconstruction for patients weighing over 74.25 kg, the operation time can surpass 15 min from the average operation time; therefore, the surgeon should be more cautious in making the ACL reconstruction procedure simple and reducing unnecessary extensions of time and not to delay or avoid surpassing the average operation time.

ACL is primarily involved in knee joint pivoting, jumping or running, and it is the most common ruptured ligament in the knee joint during sports activity. The ACL tear is one of the most common ligament injuries [3]. Numerous studies suggested that ACL-deficient knees resulted in early-onset osteoarthritis, which was associated with pain, functional limitations and decreased quality of life [36,37]. The prevalence of osteoarthritis in all adults over 45 years of age was 19.2%, whereas post-traumatic osteoarthritis following ACL injury was as high as 87% [38,39]. Not surprisingly, obesity is a significantly correlated factor with joint space narrowing following ACL injury. Obesity not only adversely affects the process of ACL reconstruction, but it also adversely affects the long-term results. Therefore, it is necessary to emphasize weight control for overweight patients after surgery.

Our study has some limitations. One of the most important limitations was that the duration from the trauma event to surgery also affected the operation time. It could have taken less operation time in the case of chronic ACL tears compared to that of acute ACL tears because it was easier to create the femoral tunnel in chronic ACL tears than in acute tears due to the absence of torn ACL remnants. In contrast, it could have taken a longer operation time in acute tears than in chronic tears because of the increasing time required to remove the torn ACL remnant because of blurred vision of the operation field due to hemarthrosis in acute tears. All patients underwent ACL reconstruction using the anteromedial portal drilling technique. Thus, it is difficult to apply or compare the results of our study to those of other studies using transtibial or external drilling techniques.

## 5. Conclusions

The weight and BMI of the pure operation time ≥61 min group were 14.7 kg and 4.5 kg/m^2^ higher, respectively, in primary ACL reconstruction with the anteromedial femoral drilling technique. The weight of patients with ACL tear was the factor, which increased the operation time for ACL reconstruction. Patients weighing over 74.25 kg were more likely to delay the ACL reconstruction operation time.

## Figures and Tables

**Table 1:** Demographic characteristics of all subjects classified as those with more than 61 min operation time (except suture time) versus less than 61 min operation time (except suture time).

	Overall (*n* = 103)	Pure OP Time ≥ 61 min (*n* = 34)	Pure OP Time < 61 min (*n* = 69)	*p*-Value
Sex (male/female)	67/36	25/9	42/27	
Age (years)	31.9 ± 12.6	31.4 ± 11.7	32.3 ± 13.1	0.76
Height (cm)	170.7 ± 8.7	172.3 ± 8.4	169.9 ± 8.7	0.2
Weight (kg)	72.4 ± 12.7	82.3 ± 10.1	67.6 ± 10.9	<0.001
Body mass index (kg/m^2^)	24.9 ± 4.1	27.9 ± 3.9	23.4 ± 3.3	0.018
OP time (min)	62.5 ± 10.6	66.4 ± 9.8	60.6 ± 10.5	
Pure OP time (min)	45.9 ± 13.4	63.3 ± 3.0	37.4 ± 6.4	

OP time: operation time, Pure OP time: pure operation time (except suture time).

**Table 2:** Correlation coefficients of operation time or pure operation time (except suture time) and demographic characteristics.

Variables	OP Time		Pure OP Time	
	Pearson Correlation Coefficient (*r*)	*p*-Value	Pearson Correlation Coefficient (*r*)	*p*-Value
Age (years)	0.104	0.3	0.013	0.89
Height (cm)	0.037	0.71	0.037	0.18
Weight (kg)	0.148	0.14	0.635	**<0.001**
Body mass index (kg/m^2^)	0.147	0.14	0.584	**<0.001**

OP time: operation time, pure OP time: pure operation time (except suture time)**;** Values in bold indicate statistical significance (*p* < 0.05).

**Table 3:** Multiple linear regression analysis to identify factors affecting pure operation time (except suture time).

		Unstandardized Coefficients		Standardized Coefficients	
Dependent Variable	Explicative Variable	B	Standard Error	b	*p*-Value
Pure operation time (except suture time)	Age (years)	−0.035	0.085	−0.033	0.68
	Height (cm)	0.948	0.784	1.21	0.23
	Weight (kg)	0.671	0.081	0.635	**<0.001**
	Body mass index (kg/m^2^)	3.91	2.741	1.195	0.16

Values in bold indicate statistical significance (*p* < 0.05).

## Data Availability

Not applicable.

## References

[1] Shankar D.S., Vasavada K.D., Avila A., DeClouette B., Aziz H., Strauss E.J., Alaia M.J., Jazrawi L.M., Gonzalez-Lomas G., Campbell K.A. (2023). Acceptable clinical outcomes despite high reoperation rate at minimum 12-month follow-up after concomitant arthroscopically assisted anterior cruciate ligament reconstruction and medial meniscal allograft transplantation. Knee Surg. Relat. Res..

[2] Morgan A.M., Bi A.S., Kaplan D.J., Alaia M.J., Strauss E.J., Jazrawi L.M. (2022). An eponymous history of the anterolateral ligament complex of the knee. Knee Surg. Relat. Res..

[3] Sundararajan S.R., Ramakanth R., Jha A.K., Rajasekaran S. (2022). Outside-in technique versus inside-out semitendinosus graft harvest technique in ACLR: A randomised control trial. Knee Surg. Relat. Res..

[4] Kim S., Bosque J., Meehan J.P., Jamali A., Marder R. (2011). Increase in outpatient knee arthroscopy in the United States: A comparison of National Surveys of Ambulatory Surgery, 1996 and 2006. J. Bone Joint Surg. Am..

[5] Gans I., Retzky J.S., Jones L.C., Tanaka M.J. (2018). Epidemiology of Recurrent Anterior Cruciate Ligament Injuries in National Collegiate Athletic Association Sports: The Injury Surveillance Program, 2004–2014. Orthop. J. Sports Med..

[6] Sherman S.L., Calcei J., Ray T., Magnussen R.A., Musahl V., Kaeding C.C., Clatworthy M., Bergfeld J.A., Arnold M.P. (2021). ACL Study Group presents the global trends in ACL reconstruction: Biennial survey of the ACL Study Group. J. ISAKOS.

[7] Belmont P.J., Goodman G.P., Hamilton W., Waterman B.R., Bader J.O., Schoenfeld A.J. (2014). Morbidity and mortality in the thirty-day period following total hip arthroplasty: Risk factors and incidence. J. Arthroplast..

[8] Belmont P.J., Goodman G.P., Waterman B.R., Bader J.O., Schoenfeld A.J. (2014). Thirty-day postoperative complications and mortality following total knee arthroplasty: Incidence and risk factors among a national sample of 15,321 patients. J. Bone Joint Surg. Am..

[9] Boddapati V., Fu M.C., Schairer W.W., Ranawat A.S., Dines D.M., Taylor S.A., Dines J.S. (2018). Increased Shoulder Arthroscopy Time Is Associated With Overnight Hospital Stay and Surgical Site Infection. Arthroscopy.

[10] Agarwalla A., Gowd A.K., Liu J.N., Garcia G.H., Bohl D.D., Verma N.N., Forsythe B. (2019). Effect of Operative Time on Short-Term Adverse Events After Isolated Anterior Cruciate Ligament Reconstruction. Orthop. J. Sports Med..

[11] Westermann R., Anthony C.A., Duchman K.R., Gao Y., Pugely A.J., Hettrich C.M., Amendola N., Wolf B.R. (2017). Infection following Anterior Cruciate Ligament Reconstruction: An Analysis of 6389 Cases. J. Knee Surg..

[12] Mont M.A., Jacobs J.J., Boggio L.N., Bozic K.J., Della Valle C.J., Goodman S.B., Lewis C.G., Yates A.J., Watters W.C., Turkelson C.M. (2011). Preventing venous thromboembolic disease in patients undergoing elective hip and knee arthroplasty. J. Am. Acad. Orthop. Surg..

[13] Bohl D.D., Ondeck N.T., Darrith B., Hannon C.P., Fillingham Y.A., Della Valle C.J. (2018). Impact of Operative Time on Adverse Events Following Primary Total Joint Arthroplasty. J. Arthroplast..

[14] Kim D.Y., Seo Y.C., Kim C.W., Lee C.R., Jung S.H. (2022). Factors affecting range of motion following two-stage revision arthroplasty for chronic periprosthetic knee infection. Knee Surg. Relat. Res..

[15] Hanly R.J., Marvi S.K., Whitehouse S.L., Crawford R.W. (2016). Morbid Obesity in Total Hip Arthroplasty: Redefining Outcomes for Operative Time, Length of Stay, and Readmission. J. Arthroplast..

[16] Mednick R.E., Alvi H.M., Krishnan V., Lovecchio F., Manning D.W. (2014). Factors Affecting Readmission Rates Following Primary Total Hip Arthroplasty. J. Bone Joint Surg. Am..

[17] Reinke C.E., Kelz R.R., Zubizarreta J.R., Mi L., Saynisch P., Kyle F.A., Even-Shoshan O., Fleisher L.A., Silber J.H. (2012). Obesity and readmission in elderly surgical patients. Surgery.

[18] Saucedo J.M., Marecek G.S., Wanke T.R., Lee J., Stulberg S.D., Puri L. (2014). Understanding readmission after primary total hip and knee arthroplasty: Who’s at risk?. J. Arthroplast..

[19] Ruopp M.D., Perkins N.J., Whitcomb B.W., Schisterman E.F. (2008). Youden Index and optimal cut-point estimated from observations affected by a lower limit of detection. Biom. J..

[20] Gadinsky N.E., Manuel J.B., Lyman S., Westrich G.H. (2012). Increased operating room time in patients with obesity during primary total knee arthroplasty: Conflicts for scheduling. J. Arthroplast..

[21] Kadry B., Press C.D., Alosh H., Opper I.M., Orsini J., Popov I.A., Brodsky J.B., Macario A. (2014). Obesity increases operating room times in patients undergoing primary hip arthroplasty: A retrospective cohort analysis. PeerJ.

[22] Raphael I.J., Parmar M., Mehrganpour N., Sharkey P.F., Parvizi J. (2013). Obesity and operative time in primary total joint arthroplasty. J. Knee Surg..

[23] Traven S.A., Wolf G.J., Goodloe J.B., Reeves R.A., Woolf S.K., Slone H.S. (2021). Elevated BMI increases concurrent pathology and operative time in adolescent ACL reconstruction. Knee Surg. Sports Traumatol. Arthrosc..

[24] Helito C.P., da Silva A.G.M., Guimaraes T.M., Sobrado M.F., Pecora J.R., Camanho G.L. (2022). Functional results of multiple revision anterior cruciate ligament with anterolateral tibial tunnel associated with anterolateral ligament reconstruction. Knee Surg. Relat. Res..

[25] Burnham J.M., Herbst E., Pauyo T., Pfeiffer T., Johnson D.L., Fu F.H., Musahl V. (2017). Technical Considerations in Revision Anterior Cruciate Ligament (ACL) Reconstruction for Operative Techniques in Orthopaedics. Oper. Tech. Orthop..

[26] World Health Organization (2000). Obesity: Preventing and Managing the Global Epidemic.

[27] Ballal M.S., Khan Y., Hastie G., Hatcher A., Coogan S., McNicholas M.J. (2013). Functional outcome of primary hamstring anterior cruciate ligament reconstruction in patients with different body mass index classes. Arthroscopy.

[28] Berg E.E. (1998). Knee joint arthroscopy in the morbidly obese. Arthroscopy.

[29] Baumgarten K.M., Carlson W.O., Watson E.S. (2011). The effect of obesity on orthopaedic conditions. S D Med..

[30] Lee D.H., Kim H.J., Ahn H.S., Bin S.I. (2016). Comparison of Femoral Tunnel Length and Obliquity Between Transtibial, Anteromedial Portal, and Outside-In Surgical Techniques in Single-Bundle Anterior Cruciate Ligament Reconstruction: A Meta-analysis. Arthroscopy.

[31] Shin Y.S., Ro K.H., Lee J.H., Lee D.H. (2013). Location of the femoral tunnel aperture in single-bundle anterior cruciate ligament reconstruction: Comparison of the transtibial, anteromedial portal, and outside-in techniques. Am. J. Sports Med..

[32] Jain G., Datt R., Mahmood A., Nag H.L., Sahu A. (2021). Anteromedial Portal Reference Technique for Femoral Tunnel Depth Measurement During Arthroscopic Anterior Cruciate Ligament Reconstruction. Cureus.

[33] Burnham J.M., Malempati C.S., Carpiaux A., Ireland M.L., Johnson D.L. (2017). Anatomic Femoral and Tibial Tunnel Placement During Anterior Cruciate Ligament Reconstruction: Anteromedial Portal All-Inside and Outside-In Techniques. Arthrosc. Tech..

[34] Lubowitz J.H. (2009). Anteromedial portal technique for the anterior cruciate ligament femoral socket: Pitfalls and solutions. Arthroscopy.

[35] McAllister D.R., Parker R.D., Cooper A.E., Recht M.P., Abate J. (1999). Outcomes of postoperative septic arthritis after anterior cruciate ligament reconstruction. Am. J. Sports Med..

[36] Lohmander L.S., Englund P.M., Dahl L.L., Roos E.M. (2007). The long-term consequence of anterior cruciate ligament and meniscus injuries: Osteoarthritis. Am. J. Sports Med..

[37] Shelbourne K.D., Stube K.C. (1997). Anterior cruciate ligament (ACL)-deficient knee with degenerative arthrosis: Treatment with an isolated autogenous patellar tendon ACL reconstruction. Knee Surg. Sports Traumatol. Arthrosc..

[38] Nakata K., Shino K., Horibe S., Tanaka Y., Toritsuka Y., Nakamura N., Koyanagi M., Yoshikawa H. (2008). Arthroscopic anterior cruciate ligament reconstruction using fresh-frozen bone plug-free allogeneic tendons: 10-year follow-up. Arthroscopy.

[39] Lawrence R.C., Felson D.T., Helmick C.G., Arnold L.M., Choi H., Deyo R.A., Gabriel S., Hirsch R., Hochberg M.C., Hunder G.G. (2008). Estimates of the prevalence of arthritis and other rheumatic conditions in the United States. Part II. Arthritis Rheum..

